# Major Adverse Kidney Events in Pediatric Continuous Kidney Replacement Therapy

**DOI:** 10.1001/jamanetworkopen.2024.0243

**Published:** 2024-02-23

**Authors:** Dana Y. Fuhrman, Erin K. Stenson, Issa Alhamoud, Rashid Alobaidi, Gabriella Bottari, Sarah Fernandez, Francesco Guzzi, Taiki Haga, Ahmad Kaddourah, Eleonora Marinari, Tahagod H. Mohamed, Catherine J. Morgan, Theresa Mottes, Tara M. Neumayr, Nicholas J. Ollberding, Valeria Raggi, Zaccaria Ricci, Emily See, Natalja L. Stanski, Huaiyu Zang, Emily Zangla, Katja M. Gist

**Affiliations:** 1 University of Pittsburgh Medical Center Children’s Hospital of Pittsburgh, Pittsburgh, Pennsylvania; 2 Children’s Hospital Colorado, University of Colorado School of Medicine, Aurora; 3 University of Iowa Stead Family Children’s Hospital, Carver College of Medicine, Iowa City; 4 University of Alberta, Edmonton, Canada; 5 Bambino Gesù Children’s Hospital, IRCCS, Rome, Italy; 6 Gregorio Marañón University Hospital, School of Medicine, Madrid, Spain; 7 Santo Stefano Hospital, Prato, Italy; 8 Osaka City General Hospital, Osaka, Japan; 9 Sidra Medicine, Doha, Qatar; 10 Weill Cornell Medical College, Ar-Rayyan, Qatar; 11 Nationwide Children’s Hospital, The Heart Center, The Ohio State University College of Medicine, Columbus; 12 University of Alberta, Edmonton, Alberta, Canada; 13 Ann and Robert H. Lurie Children’s Hospital of Chicago, Chicago, Illinois; 14 Washington University School of Medicine, St Louis Children’s Hospital, St Louis, Missouri; 15 Cincinnati Children’s Hospital Medical Center; University of Cincinnati College of Medicine, Cincinnati, Ohio; 16 Meyer Children's Hospital, IRCCS, Florence, Italy; 17 Royal Children’s Hospital, University of Melbourne, Murdoch Children’s Research Institute, Melbourne, Victoria, Australia; 18 University of Minnesota, Minneapolis

## Abstract

**Importance:**

Continuous kidney replacement therapy (CKRT) is increasingly used in youths with critical illness, but little is known about longer-term outcomes, such as persistent kidney dysfunction, continued need for dialysis, or death.

**Objective:**

To characterize the incidence and risk factors, including liberation patterns, associated with major adverse kidney events 90 days after CKRT initiation (MAKE-90) in children, adolescents, and young adults.

**Design, Setting, and Participants:**

This international, multicenter cohort study was conducted among patients aged 0 to 25 years from The Worldwide Exploration of Renal Replacement Outcomes Collaborative in Kidney Disease (WE-ROCK) registry treated with CKRT for acute kidney injury or fluid overload from 2015 to 2021. Exclusion criteria were dialysis dependence, concurrent extracorporeal membrane oxygenation use, or receipt of CKRT for a different indication. Data were analyzed from May 2 to December 14, 2023.

**Exposure:**

Patient clinical characteristics and CKRT parameters were assessed. CKRT liberation was classified as successful, reinstituted, or not attempted. Successful liberation was defined as the first attempt at CKRT liberation resulting in 72 hours or more without return to dialysis within 28 days of CKRT initiation.

**Main Outcomes and Measures:**

MAKE-90, including death or persistent kidney dysfunction (dialysis dependence or ≥25% decline in estimated glomerular filtration rate from baseline), were assessed.

**Results:**

Among 969 patients treated with CKRT (529 males [54.6%]; median [IQR] age, 8.8 [1.7-15.0] years), 630 patients (65.0%) developed MAKE-90. On multivariable analysis, cardiac comorbidity (adjusted odds ratio [aOR], 1.60; 95% CI, 1.08-2.37), longer duration of intensive care unit admission before CKRT initiation (aOR for 6 days vs 1 day, 1.07; 95% CI, 1.02-1.13), and liberation pattern were associated with MAKE-90. In this analysis, patients who successfully liberated from CKRT within 28 days had lower odds of MAKE-90 compared with patients in whom liberation was attempted and failed (aOR, 0.32; 95% CI, 0.22-0.48) and patients without a liberation attempt (aOR, 0.02; 95% CI, 0.01-0.04).

**Conclusions and Relevance:**

In this study, MAKE-90 occurred in almost two-thirds of the population and patient-level risk factors associated with MAKE-90 included cardiac comorbidity, time to CKRT initiation, and liberation patterns. These findings highlight the high incidence of adverse outcomes in this population and suggest that future prospective studies are needed to better understand liberation patterns and practices.

## Key Points

**Question:**

How often do major adverse kidney events (death, dialysis, or persistent kidney dysfunction) at 90 days (MAKE-90) occur in youths treated with continuous kidney replacement therapy (CKRT), and what are the risk factors associated with MAKE-90 in youths treated with CKRT?

**Findings:**

In this cohort study of 969 patients treated with CKRT aged 0 to 25 years, MAKE-90 occurred in 65.0% of patients. Risk factors associated with increased odds of MAKE-90 included failure to liberate from CKRT and cardiac comorbidity.

**Meaning:**

This study found that MAKE-90 occurred commonly in youths treated with CKRT and identified patient-level risk factors associated with MAKE-90, highlighting the importance of CKRT liberation.

## Teaser

This cohort study examines incidence of major adverse kidney events at 90 days and risk factors associated with them among youths treated with continuous kidney replacement therapy.

## Introduction

Children, adolescents, and young adults with critical illness have a high likelihood of developing acute kidney injury (AKI) and disorders of fluid balance, including pathologic fluid overload.^1^ Patients with severe AKI and fluid overload are commonly treated with continuous kidney replacement therapy (CKRT) to allow for electrolyte, acid base, and fluid balance optimization. Youths treated with CKRT represent one of the most critically ill populations treated in intensive care units (ICUs). While there is significant literature highlighting the short-term morbidity and mortality, the incidence and risk factors associated with modern consensus composite outcomes that go beyond mortality have not been described in youths treated with CKRT, to our knowledge.

To standardize the reporting of cohort studies and clinical trials in critical care nephrology, the National Institute of Diabetes and Digestive Kidney Diseases workgroup on clinical trials put forth the major adverse kidney events at 90 days (MAKE-90) composite outcome. The power of the MAKE-90 outcome is that it expands outcome reporting from mortality alone to include patient-centered end points (new kidney replacement therapy and persistent kidney dysfunction).^2,3^ While MAKE-90 has been studied in adults treated with CKRT, it has yet to be studied in pediatric patients, to our knowledge.^4^ A better understanding of the incidence of MAKE-90 and patient-level risk factors associated with MAKE-90 will be a critical step to improve clinical care, develop future clinical trials, and ultimately improve outcomes. Although large studies from 2017 to 2021^5,6,7^ have aimed to investigate the optimal time to initiate CKRT, there is no consensus on when to trial CKRT liberation. In adults with critically illness, failure to successfully liberate from CKRT is associated with an increased risk of mortality, dialysis dependence, and continued kidney dysfunction, all components of MAKE-90.^4,8,9^

To begin to address these knowledge gaps, we performed a planned analysis of the Worldwide Exploration of Renal Replacement Outcomes Collaborative in Kidney Disease (WE-ROCK) registry of more than 900 youths treated with CKRT. The aims of this study were to describe the incidence of MAKE-90, identify independent patient clinical characteristics, and identify independent CKRT parameters (including liberation patterns) associated with MAKE-90 in youths treated with CKRT for AKI or fluid overload. We hypothesized that the incidence of MAKE-90 would be high in this population and that there would be patient-level risk factors (ie, primary comorbidities, reason for hospital admission, and illness severity) and CKRT parameters (ie, duration, timing of initiation, and liberation pattern) associated with MAKE-90.

## Methods

Each participating site in this cohort study sought institutional board approval, with a waiver of informed consent or parental permission due to the retrospective nature of the study. We followed the Strengthening the Reporting of Observational Studies in Epidemiology (STROBE) guideline for reporting study results.

### Study Design and Setting

Data for the study were collected from the WE-ROCK of pediatric and young adult patients who required CKRT. WE-ROCK was formed to create an international registry of patients receiving CKRT from 2015 to 2021, with a minority of patients included from 2015 to 2017 (67 patients). The registry includes patients from 32 centers and 7 nations.

### Participants

Patients were included if they were aged 0 and 25 years and required CKRT due to AKI or fluid overload. Patients with previous dialysis dependence, extracorporeal membrane oxygenation use, or receipt of CKRT for a different indication were excluded. Detailed methods have been previously described.^10^

### Data Collection

A retrospective electronic health record review was performed, and data were entered into a central research electronic data capture (REDCap) database. Reference creatinine level was defined as the lowest serum creatinine level within 90 days prior to admission. If no serum creatinine was available prior to hospital admission, we imputed an estimated glomerular filtration rate (eGFR) of 100 ml/min/1.73 m^2^ and back calculated a reference serum creatinine level based on the bedside Schwartz equation, as previously described.^11,12^

### Liberation Definitions

Patients were categorized into 1 of 3 liberation categories based on the first liberation attempt during the first 28 days of CKRT. Only the first attempt at liberation was considered: (1) liberated: patients had no receipt of CKRT or other dialysis modality for 72 or more hours after discontinuing CKRT. (2) Reinstituted: patients resumed CKRT or another dialysis modality within 72 hours of a liberation attempt. (3) Not attempted: patients had no attempt at CKRT liberation within the first 28 days after CKRT initiation. A period of 72 hours has been used in numerous other investigations assessing kidney recovery as an adequate period to capture successful liberation and return of intrinsic kidney function.^4,13,14,15^

### Outcomes

Our primary outcome was MAKE-90. Based on previous studies, we defined MAKE-90 as a binary, composite outcome that includes any of the following: persistent kidney dysfunction, defined as a 25% or greater decline in eGFR from the reference value at 90 days; continued need for any form of KRT at 90 days; and mortality, defined as death from any cause at 90 days.^2^ We chose a 90-day period in accordance with the Kidney Disease: Improving Global Outcomes (KDIGO) guidelines, which define chronic kidney disease as kidney dysfunction lasting at least 90 days.^16^ If a patient died, the death end point was met but not persistent kidney dysfunction or dialysis dependence. If a patient met the dialysis end point, the individual was also considered to have persistent kidney dysfunction.^17^ Our secondary outcome was mortality.

### Statistical Analysis

Continuous data are reported as medians with IQRs, while categorical data are reported as frequencies with percentages. We compared baseline characteristics between patients with and without MAKE-90 using the Wilcoxon rank-sum and χ^2^ test for continuous and categorical variables, respectively. Kaplan-Meier curves were generated to depict the cumulative probability of death over 90 days for the overall cohort and for each liberation pattern group. The log-rank test was used to compare mortality differences between groups. Time zero was the day of CKRT initiation. Patients were censored if they did not experience death within 90 days of CKRT liberation.

To assess the association of patient clinical characteristics and CKRT parameters with the development of MAKE-90, a multivariable logistic regression model was used to estimate adjusted odds ratios (aORs) and 95% CIs for MAKE-90. A priori relevant covariates were selected based on the existing literature and clinical relevance, which included age, sex, reference creatinine level, race (reported by WE-ROCK investigators), ethnicity (reported by WE-ROCK investigators), comorbidities, admission weight, sepsis, reason for admission, Pediatric Risk of Mortality (PRISM) III score, vasoactive-inotropic score (VIS), Pediatric Logistic Organ Dysfunction-2 (PELOD-2) score, percentage cumulative fluid balance, urine output, CKRT duration, time from ICU admission to CKRT initiation, median calculated CKRT dose, and liberation pattern. We collected data on race and ethnicity due to possible differences in outcomes. Race options included American Indian, Asian or Pacific Islander, Black, White, more than 1 race, and unknown or not disclosed. Ethnicity options included Hispanic or Latino and not Hispanic or Latino. An interaction term between liberation pattern and cardiac comorbidities was considered based on subject matter knowledge but not retained in final models owing to the lack of an association. Robust standard errors computed via the Huber-White method were used to correct for the clustering of patients within hospitals. For continuous covariates, linear associations with the outcome were assumed and aORs are presented as interquartile odds ratios comparing a reference (25th percentile) with a contrast (75th percentile) value. The model performance was measured by optimism-corrected *C* index and Brier score. A calibration curve of the observed vs estimated probability was used to internally validate the accuracy of estimations using 1000 bootstrap resamplings. Sensitivity analysis was performed using similar methods to examine risk factors associated with persistent kidney dysfunction or dialysis dependence at 90 days.

In all analyses, a 2-sided *P* value < .05 was considered statistically significant. All statistical analyses were performed using R statistical software version 4.3.1 (R Project for Statistical Computing). The rms package version 6.7.1 was used to perform logistic regression, model validation, and calibration. Kaplan-Meier curves were generated using survival version 3.5.5 and survminer version 0.4.9 packages, and tables were generated using the gtsummary package version 1.7.2. The analysis was conducted from May 2 to December 14, 2023.

## Results

### Incidence of MAKE-90

**Figure 1.**
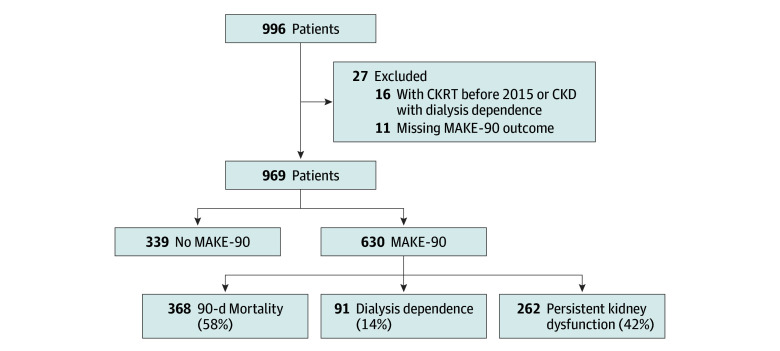
Study Flowchart CKRT indicates continuous kidney replacement therapy; CKD, chronic kidney disease; MAKE-90, major adverse kidney events (including death, dialysis, or persistent kidney dysfunction) at 90 days.

A total of 969 patients (529 males [54.6%]; median [IQR] age, 8.8 [1.7-15.0] years; 16 American Indian [1.9%], 40 Asian or Pacific Islander [4.7%], 127 Black [14.9%], and 652 White [76.4%]; 160 Hispanic [18.6%]) were included in this study (Figure 1). Demographics of the overall cohort and patients with and without MAKE-90 outcomes are shown in Table 1. MAKE-90 occurred in 630 patients (65.0%). Most patients with MAKE-90 fulfilled criteria via mortality (368 patients [58.4%]), while 91 patients (14.4%) were dialysis dependent and 262 patients (41.6%) had persistent kidney dysfunction; among all patients in the study, rates were 38.0% for mortality, 9.4% for dialysis dependence, and 27.0% for persistent kidney dysfunction (Figure 1). A Kaplan-Meier mortality curve over 90 days for the overall cohort is presented in eFigure 1 in Supplement 1.

**Table 1.** Patient Characteristics and CKRT Parameters

Variable	Patients, No. (%)			*P* value^a^
	Overall (N = 969)	No MAKE-90 (n = 339)	MAKE-90 (n = 630)	
Patient baseline characteristic				
Age, median (IQR), y	8.8 (1.7-15.0)	6.8 (2.0-14.0)	8.3 (1.4-15.6)	.32
Sex				
Male	529 (54.6)	190 (56.0)	339 (53.8)	.55
Female	440 (45.4)	149 (44.0	291 (46.2)	
Reference serum creatinine level, median (IQR), mg/dL^b^	0.4 (0.3-0.7)	0.5 (0.3-0.7)	0.4 (0.2-0.6)	<.001
Race				
American Indian	16 (1.9)	3 (1.0)	13 (2.4)	.53
Asian or Pacific Islander	40 (4.7)	15 (4.9)	25 (4.6)	
Black	127 (14.9)	51 (16.6)	76 (13.9)	
White	652 (76.4)	232 (75.6)	420 (76.9)	
≥1 Race^c^	18 (2.1)	6 (2.0)	12 (2.2)	
Ethnicity				
Hispanic or Latino	160 (18.6)	48 (15.9)	112 (20.1)	.16
Not Hispanic or Latino	700 (81.4)	254 (84.1)	446 (79.9)	
Comorbidities				
None	192 (19.8)	107 (31.6)	85 (13.5)	<.001
Primary category				
Cardiac	192 (19.8)	47 (13.9)	145 (23.0)	<.001
Endocrinologic	62 (6.4)	24 (7.1)	38 (6.0)	.62
Gastrointestinal	184 (19.0)	63 (18.6)	121 (19.2)	.88
Hematologic	124 (12.8)	37 (10.9)	87 (13.8)	.24
Immunologic	153 (15.8)	30 (8.8)	123 (19.5)	<.001
Neurologic	131 (13.5)	43 (12.7)	88 (14.0)	.65
Nephrologic or urologic	91 (9.4)	26 (7.7)	65 (10.3)	.22
Oncologic	220 (22.7)	59 (17.4)	161 (25.6)	.005
Respiratory	133 (13.7)	41 (12.1)	92 (14.6)	.32
Admission characteristic				
Admission weight, median (IQR), kg	26.8 (11.6-54.9)	25.4 (12.1-55.5)	27.8 (11.0-54.4)	.51
Sepsis at ICU admission	442 (45.6)	143 (42.2)	299 (47.5)	.13
Admission category				
Shock, infection, or major trauma	360 (37.2)	154 (45.4)	206 (32.7)	<.001
Respiratory failure	193 (19.9)	33 (9.7)	160 (25.4)	
Postsurgical or minor trauma	45 (5.0)	19 (5.6)	29 (4.6)	
Central nervous system dysfunction	37 (3.8)	10 (2.9)	27 (4.3)	
Pain or sedation management	8 (0.8)	3 (0.9)	5 (0.8)	
Primary cardiac: congenital	31 (3.2)	6 (1.8)	25 (4.0)	
Primary cardiac: postsurgical	49 (5.1)	18 (5.3)	31 (4.9)	
Primary cardiac: cardiomyopathy	39 (4.0)	12 (3.5)	27 (4.3)	
Other	204 (21.1)	84 (24.8)	120 (19.0)	
Illness severity parameter, median (IQR)				
PRISM III score at ICU admission	14 (10-18)	14 (10-18)	14 (10-18)	.47
VIS at CKRT initiation	5 (0-20)	2.5 (0-15)	5 (0-20)	.01
Missing	2 (.2)	0 (0)	2 (.6)	NA
VIS at CKRT liberation attempt	0 (0-3.1)	0 (0-2.0)	0 (0-5)	.003
PELOD-2 score at CKRT initiation	7 (4-9)	6 (4-8)	7 (5-10)	<.001
PELOD-2 score at CKRT liberation attempt	5 (3-7)	5 (3-7)	5 (2-7)	.12
Percentage cumulative fluid balance (ICU admission to CKRT initiation)	7.4 (2.4-18.1)	7.2 (2.1-16.2)	7.7 (2.5-19.9)	.20
Urine output 24 h before CKRT initiation, mL/kg/h	0.5 (0.1-1.2)	0.5 (0.2-1.4)	0.5 (0.1-1.2)	.14
Missing	8 (.8)	4 (1.2)	4 (.6)	NA
CKRT parameter				
CKRT duration, median (IQR), d	6 (3-14)	5 (3-10)	8 (3-18)	<.001
Time from ICU admission to CKRT initiation, median (IQR), d	2 (1-6)	2 (1-4)	3 (1-8)	.002
Missing	1 (.1)	0 (0)	1 (.2)	NA
Calculated CKRT dose, median (IQR), mL/kg/h	43 (32-60)	44 (32-60)	43 (32-61)	.61
Missing	25 (2.6)	9 (26.5)	16 (25.4)	NA
Liberation pattern				
Liberated	328 (33.8)	218 (64.3)	110 (17.5)	<.001
Reinstituted	288 (29.7)	109 (32.2)	179 (28.4)	
Not attempted	353 (36.4)	12 (3.5)	341 (54.1)	

Abbreviations: CKRT, continuous kidney replacement therapy; ICU, intensive care unit; MAKE-90, major adverse kidney events (including death, dialysis, or persistent kidney dysfunction) at 90 days; NA, not applicable; PELOD-2, Pediatric Logistic Organ Dysfunction-2; PRISM, Pediatric Risk of Mortality; VIS, vasoactive-inotropic score.

SI conversion factor: To convert creatinine to micromoles per liter, multiply by 88.4.

^a^ *P* values calculated using χ^2^ test or Wilcoxon rank sum test.

^b^ The lowest serum creatinine level within 90 days prior to admission or an imputed serum creatinine from an estimated glomerular filtration rate of 100 ml/min/1.73 m^2^.

^c^ Respondents could choose more than 1 race.

### Risk Factors Associated With MAKE-90

Patients with no prior comorbidities constituted the smallest proportion of the population with MAKE-90 (85 patients [13.5%]), while patients with cardiac (145 patients [23.0%]), oncologic (161 patients [25.6%]), or immunologic (123 patients [19.5%]) comorbidities constituted the largest proportions of the MAKE-90 population. Patients meeting MAKE-90 criteria had higher median (IQR) illness severity scores at CKRT initiation than patients not meeting the criteria, including VIS (5 [0-20] vs 2.5 [0-15]) and PELOD-2 score (7 [5-10] vs 6 [4-8]). Patients who developed MAKE-90 had a longer time between ICU admission and CKRT initiation and a longer CKRT duration compared with those who did not develop MAKE-90 (Table 1).

**Figure 2.**
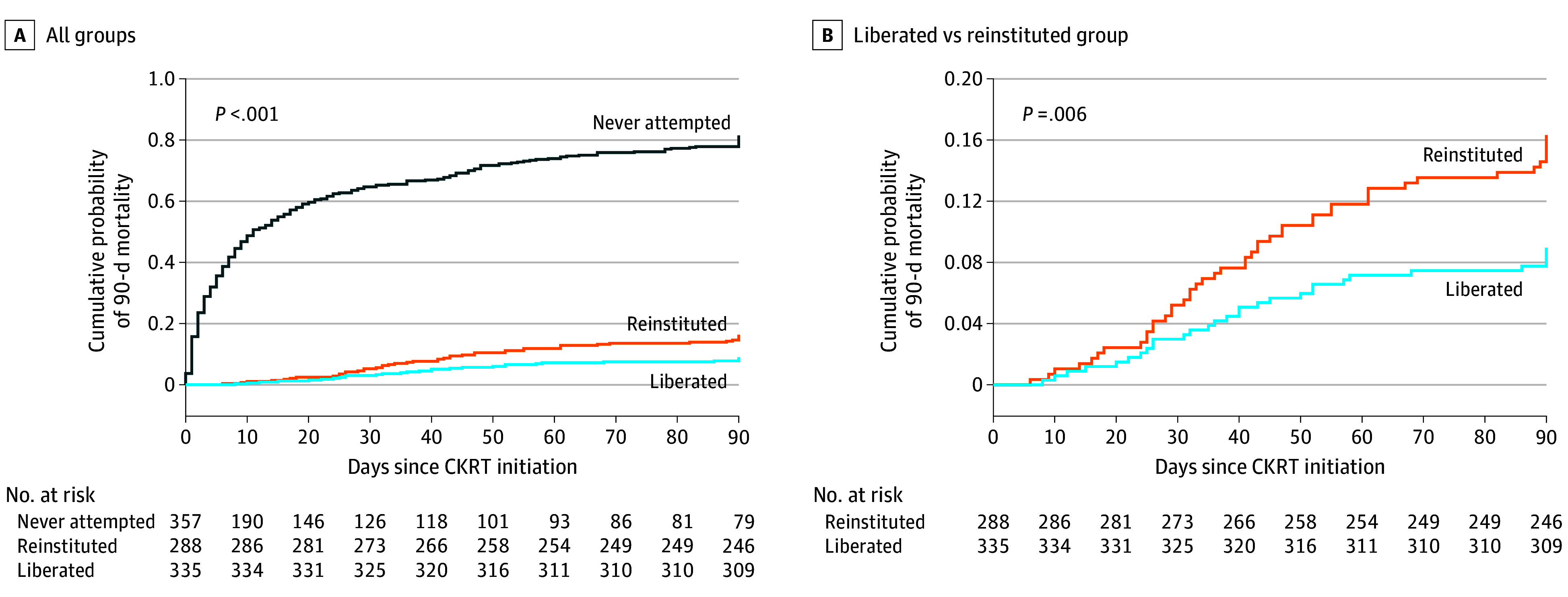
Kaplan-Meier Curves for Mortality A, The Kaplan-Meier mortality curve within 90 days for the 3 groups is shown. Time 0 is the day of continuous kidney replacement therapy (CKRT) initiation. At 90 days after CKRT initiation, there was a statistically significant mortality difference among patients in the liberated (26 of 335 patients [7.8%]), reinstituted (42 of 288 patients [14.6%]), and not attempted (278 of 357 patients [77.9%]) groups (log-rank *P* < .001). B, This difference persisted when comparing the liberated group with the reinstituted group (log-rank *P* = .006).

The median (IQR) time of liberation was 4 (2-7) days for the group that successfully liberated and 9 (4-17) days for the group that required reinstitution of CKRT. Compared with 288 patients (29.4%) who required reinstitution of CKRT or 335 patients (34.2%) who were successfully liberated, outcomes were overall worse in 357 patients (36.4%) among whom liberation was not attempted within 28 days, including greater ICU and hospital mortality, longer duration of ICU stay, and longer duration of mechanical ventilation (eTable 1 in Supplement 1). Rates of MAKE-90 differed among the liberated group (110 patients [17.5%]), reinstituted group (179 patients [28.4%]), and group in which liberation was not attempted (341 patients [54.1%]) (Table 1). At 90 days, there was a statistically significant mortality difference comparing patients who successfully liberated (26 patients [7.8%]), required reinstitution of CKRT after a liberation attempt (42 patients [14.6%]), and in whom no liberation attempt was trialed (278 patients [77.9%]) (*P* < .001) (Figure 2A). There was also a significant difference in mortality comparing the patient group that successfully liberated with the group that had CKRT reinstituted (log-rank *P* = .006) (Figure 2B). Patients who successfully liberated had the lowest probability of MAKE-90 (33.6%; 95% CI, 25.6%-42.7%) compared with not attempted (95.6%; 95% CI, 93.3%-97.5%) and reinstituted (61.0%; 95% CI, 51.2%-69.9%) groups (eFigure 2 in Supplement 1).

### Independent Factors Associated With MAKE-90

There was no association between calendar year of treatment and MAKE-90 in the primary outcome model; thus, this variable was not included in final models. The multivariable regression model to estimate odds of MAKE-90 is shown in Table 2. After 34 patients with missing covariate data (3.5%) were excluded, the model was conducted among 935 patients. It had good discrimination and accuracy in estimating, with an optimism-corrected *C* index of 0.84 and a Brier score of 0.15. The calibration curve confirmed that the model’s estimated probabilities for MAKE-90 were in line with observations (eFigure 3 in Supplement 1). Patients who were successfully liberated had 68% lower odds of meeting MAKE-90 criteria (aOR, 0.32; 95% CI, 0.22-0.48) compared with those in whom CKRT was reinitiated after a liberation attempt. Furthermore, patients who were successfully liberated had 98% lower odds of meeting criteria for MAKE-90 compared with patients in whom liberation was never attempted (aOR, 0.02; 95% CI, 0.01-0.04).

**Table 2.** Multivariable Regression Model Estimating Odds of MAKE-90 (N = 935)^a^

Variable	Reference	Contrast	aOR (95% CI)^b^
Liberation pattern^c^			
Liberated vs reinstituted	Reinstituted	Liberated	0.32 (0.22-0.48)
Liberated vs not attempted	Not attempted	Liberated	0.02 (0.01-0.04)
No comorbidities	No	Yes	0.48 (0.30-0.76)
Primary comorbidity: cardiac	No	Yes	1.60 (1.08-2.37)
Primary comorbidity: nephrologic or urologic	No	Yes	1.26 (0.66-2.40)
Primary comorbidity: immunologic	No	Yes	1.67 (0.92-3.04)
Primary comorbidity: oncologic	No	Yes	1.19 (0.73-1.93)
Sepsis at ICU admission	No	Yes	0.91 (0.58-1.41)
VIS at CKRT initiation	0	20	0.92 (0.79-1.07)
PELOD-2 score at CKRT initiation	4	9	1.21 (0.90-1.63)
Percentage cumulative fluid balance (ICU admission to CKRT initiation)	2.4	18.1	1.05 (0.97-1.14)
Time from ICU admission to CKRT initiation, d	1	6	1.07 (1.02-1.13)
Calculated CKRT dose, median (IQR), mL/kg/h	32	60.1	1.05 (0.95-1.22)
CKRT duration, d	3	14	1.09 (0.95-1.24)
Urine output 24 h prior to CKRT initiation, mL/kg/h	0.1	1.2	0.88 (0.77-1.01)

Abbreviations: aOR, adjusted odds ratio; CKRT, continuous kidney replacement therapy; ICU, intensive care unit; MAKE-90, major adverse kidney events (including death, dialysis, or persistent kidney dysfunction) at 90 days; PELOD-2, Pediatric Logistic Organ Dysfunction-2; VIS, vasopressor-inotropic score.

^a^ A total of 34 patients were not included in the analysis due to missing covariate data.

^b^ The aORs and 95% CIs were obtained by logistic regression; aORs for continuous factors are scaled to reflect the IQR OR (ie, reference = 25th percentile and contrast = 75th percentile).

^c^ The same model was rerun with each liberation pattern individually with no change in covariate aORs.

A longer time from ICU admission to CKRT initiation was independently associated with an increased odds of MAKE-90 (aOR for 6 days vs 1 day, 1.07; 95% CI, 1.02-1.13) (Table 2). Having a cardiac comorbidity was associated with 1.60-fold increased odds of MAKE-90 (aOR, 1.60; 95% CI, 1.08-2.37). The absence of any comorbidity was protective against MAKE-90 (aOR, 0.48; 95% CI, 0.30-0.76). As shown in eTable 2 in Supplement 1, there were decreased odds of persistent kidney dysfunction or dialysis dependence in patients who were successfully liberated compared with those among whom CKRT was reinstituted and those in whom liberation was not attempted. The absence of comorbidities was protective against persistent kidney dysfunction or continued need for dialysis (eTable 2 in Supplement 1).

## Discussion

This cohort study found that MAKE-90 outcomes were common and occurred in almost two-thirds of youths requiring CKRT. To our knowledge, this is the first study to characterize MAKE in a multicenter cohort exclusive to children, adolescents, and young adults receiving CKRT. In contrast to investigations and consensus guidelines focused on outcomes associated with ventilator liberation in patients with acute lung injury, there is a paucity of data regarding patient outcomes related to CKRT discontinuation.^18^ This study identified patient level-factors associated with MAKE-90, including the novel description of the association of CKRT liberation patterns with MAKE-90. We found that patients who successfully liberated from CKRT within the first 28 days after starting therapy had significantly greater odds of survival and avoidance of other adverse kidney events at 90 days.

While we confirmed that youths treated with CKRT were at high risk of mortality at 90 days (368 patients [38.0%]), the power in this study is in describing the high burden of patient-level kidney outcomes in this population. We found that 262 patients (27.0%) had persistent kidney dysfunction and 91 patients (9.4%) continued to be treated with KRT at 90 days. These data may serve to provide clinicians with information to counsel families. Furthermore, these data may be used to design clinical trials and develop standardized follow-up clinics for these patients at increased risk.

To improve outcomes in youths treated with CKRT, it is important to understand risk factors associated with MAKE-90 to identify high-risk populations and potential targets for intervention. We identified patient (eg, comorbidities) and CKRT (eg, time to initiation and liberation pattern) characteristics associated with adverse kidney outcomes 90 days after CKRT initiation. It is known that AKI is common in patients with congenital heart disease and is associated with their morbidity and mortality.^19,20^ In this study, we found the highest odds of MAKE-90 outcomes in patients with cardiac comorbidities. We found that patients admitted for congenital heart disease or cardiomyopathy had the highest rates of MAKE-90. Further investigation into this cohort is warranted to identify modifiable factors associated with MAKE-90.

The optimal timing of CKRT initiation remains a controversial topic in critical care nephrology across age groups. Interestingly, we found that a longer time to CKRT initiation anchored to ICU admission was independently associated with MAKE-90. There was a 7% increase in the odds of MAKE-90 for patients who started CKRT on ICU day 6 compared with those who were initiated on ICU day 1. These findings require further exploration. Might there be a clinical phenotype that should be explored to better determine which patients will benefit from earlier compared with later CKRT initiation? Results from the Standard vs Accelerated Initiation of Renal Replacement Therapy in Acute Kidney Injury (STARRT-AKI) trial^21^ in adults support not starting CKRT until it is absolutely indicated. Further exploration of this registry is necessary to speculate rationale for the association.

While significant time and effort have been spent evaluating CKRT initiation timing,^6,7,21,22^ little is known about liberation patterns, practices, or the association of liberation attempts with outcomes. Notably, the worst outcomes in our study cohort occurred in patients in whom liberation was never attempted. Given our study findings, it is important to recognize that prolonged KRT is associated with adverse events, such as intradialytic hypotension, complications of anticoagulation, increased risk of infection, nutritional deficiencies, and increased health care cost.^3,23,24,25^ KRT may also impair kidney recovery after AKI.^26^ In our study, 33.8% of patients were successfully liberated and fulfilled the criteria of achieving more than 72 hours without KRT after therapy discontinuation. In a single-center study examining outcomes associated with CKRT liberation in adults that similarly used a 72-hour observation period, Liu and colleagues^4^ also reported a low occurrence of liberation success. It is quite possible that the high rate of liberation failure seen in both studies reflects variations in clinical practice. Currently, there are no consensus guidelines providing recommendations for clinicians deciding on the optimal timing of CKRT discontinuation. In a 2022 cross-sectional survey^27,28^ of intensivists and nurses working in 20 European pediatric ICUs, investigators reported no consensus regarding decisions on how or when to liberate patients from CKRT. Our study highlights the need for a shift in the paradigm of how we study CKRT in youths, from focusing on CKRT initiation to a more wholistic approach systematically evaluating liberation.

Our study has several notable strengths. Results are representative of a contemporary, multicenter, international cohort of pediatric patients with CKRT and no prior history of dialysis dependence. To our knowledge, this is the first study in pediatrics and one of few studies overall to examine the association of liberation from CKRT with clinically meaningful outcomes. Data collection occurred over a relatively short period, decreasing the likelihood of center practice changes.

### Limitations

This study has several limitations. Although we controlled for severity of illness, we recognize that it is possible that liberation pattern does not directly contribute to MAKE but instead is a consequence of disease severity. Alternatively, it is also feasible that pathophysiologic mechanisms contributing to adverse outcomes after CKRT are independent of severity of illness. This study is retrospective, and the data were self-reported by each participating center, which may have led to selection bias. Although we included multiple markers of severity of illness in the analysis, it is possible that residual confounding remained. Given that only the first liberation attempt was documented, we may have missed patients who successfully liberated after a subsequent liberation attempt. Although smaller centers were included in the analysis, all included hospitals were tertiary or quaternary, possibly limiting generalizability to resource-limited settings. In addition, the type I error rate may have exceeded the nominal level given that no adjustments for multiple testing were performed. The definition of liberation status may have introduced periods of immortal time in which patients could not experience the outcome, resulting in some expected bias in our estimates. Additionally, only multivariable results using complete data are reported, and 34 patients with missing data were excluded from these analyses. However, the overall proportion of missing data was less than 5%.

## Conclusions

In this cohort study, we found that MAKE were common 90 days after the initiation of CKRT in children, adolescents, and young adults. We found that successful liberation from therapy within 28 days was associated with lower odds of MAKE-90. Our study findings further suggest that cardiac diagnoses and increased duration between ICU admission and CKRT initiation contribute to MAKE at 90 days. Our study results support the need for future prospective studies exploring a causative relationship between CKRT parameters and clinically relevant outcomes in children, adolescents, and young adults.

## Notes

Supplement 1. **eTable 1.** Patient Outcomes Comparing 3 Liberation Patterns **eTable 2.** Multivariable Regression Model Estimating Persistent Kidney Dysfunction or Dialysis at 90 d **eFigure 1.** Kaplan-Meier Curve Summarizing Probability of Death Over 90 d for Overall Cohort **eFigure 2.** Estimated Probability of Meeting Primary Outcome Criteria by Liberation Pattern From Logistic Regression Model **eFigure 3.** Calibration Curves and Estimation Accuracy Measures for Logistic Regression Models of Primary Outcome

Supplement 2. **Nonauthor Collaborators.** WE-ROCK Investigators

Supplement 3.
Data Sharing Statement

## References

[1] Kaddourah A, Basu RK, Bagshaw SM, Goldstein SL; AWARE Investigators. Epidemiology of acute kidney injury in critically ill children and young adults. N Engl J Med. 2017;376(1):11-20. doi:10.1056/NEJMoa1611391 27959707 PMC5322803

[2] Billings FT IV, Shaw AD. Clinical trial endpoints in acute kidney injury. Nephron Clin Pract. 2014;127(1-4):89-93. doi:10.1159/000363725 25343828 PMC4480222

[3] Palevsky PM, Baldwin I, Davenport A, Goldstein S, Paganini E. Renal replacement therapy and the kidney: minimizing the impact of renal replacement therapy on recovery of acute renal failure. Curr Opin Crit Care. 2005;11(6):548-554. doi:10.1097/01.ccx.0000179936.21895.a3 16292058

[4] Liu C, Peng Z, Dong Y, . Continuous renal replacement therapy liberation and outcomes of critically ill patients with acute kidney injury. Mayo Clin Proc. 2021;96(11):2757-2767. doi:10.1016/j.mayocp.2021.05.031 34686364

[5] Wald R, Bagshaw SM; START-AKI Investigators. Timing of initiation of renal-replacement therapy in acute kidney injury: reply. N Engl J Med. 2020;383(18):1797-1798. 33113308 10.1056/NEJMc2027489

[6] Bhatt GC, Das RR. Early versus late initiation of renal replacement therapy in patients with acute kidney injury-a systematic review & meta-analysis of randomized controlled trials. BMC Nephrol. 2017;18(1):78. doi:10.1186/s12882-017-0486-9 28245793 PMC5331682

[7] Pan HC, Chen YY, Tsai IJ, . Accelerated versus standard initiation of renal replacement therapy for critically ill patients with acute kidney injury: a systematic review and meta-analysis of RCT studies. Crit Care. 2021;25(1):5. doi:10.1186/s13054-020-03434-z 33402204 PMC7784335

[8] Allegretti AS, Steele DJ, David-Kasdan JA, Bajwa E, Niles JL, Bhan I. Continuous renal replacement therapy outcomes in acute kidney injury and end-stage renal disease: a cohort study. Crit Care. 2013;17(3):R109. doi:10.1186/cc12780 23782899 PMC4057378

[9] Jeon J, Kim DH, Baeg SI, . Association between diuretics and successful discontinuation of continuous renal replacement therapy in critically ill patients with acute kidney injury. Crit Care. 2018;22(1):255. doi:10.1186/s13054-018-2192-9 30305122 PMC6180655

[10] Menon S, Krallman KA, Arikan AA, ; WE-ROCK Investigators. Worldwide exploration of renal replacement outcomes collaborative in kidney disease (WE-ROCK). Kidney Int Rep. 2023;8(8):1542-1552. doi:10.1016/j.ekir.2023.05.026 37547524 PMC10403688

[11] Joyce EL, DeAlmeida DR, Fuhrman DY, Priyanka P, Kellum JA. eResearch in acute kidney injury: a primer for electronic health record research. Nephrol Dial Transplant. 2019;34(3):401-407. doi:10.1093/ndt/gfy052 29617846 PMC6399481

[12] Schwartz GJ, Haycock GB, Edelmann CM Jr, Spitzer A. A simple estimate of glomerular filtration rate in children derived from body length and plasma creatinine. Pediatrics. 1976;58(2):259-263. doi:10.1542/peds.58.2.259 951142

[13] Yoshida T, Matsuura R, Komaru Y, . Kinetic estimated glomerular filtration rate as a predictor of successful continuous renal replacement therapy discontinuation. Nephrology (Carlton). 2019;24(3):287-293. doi:10.1111/nep.13396 29717547

[14] Katayama S, Uchino S, Uji M, ; Japanese Society of Education for Physicians and Trainees in Intensive Care (JSEPTIC) Clinical Trial Group. Factors predicting successful discontinuation of continuous renal replacement therapy. Anaesth Intensive Care. 2016;44(4):453-457. doi:10.1177/0310057X1604400401 27456174

[15] Stads S, Kant KM, de Jong MFC, . Predictors of 90-day restart of renal replacement therapy after discontinuation of continuous renal replacement therapy, a prospective multicenter study. Blood Purif. 2019;48(3):243-252. doi:10.1159/000501387 31330511 PMC6878749

[16] Kellum JA, Sileanu FE, Bihorac A, Hoste EA, Chawla LS. Recovery after acute kidney injury. Am J Respir Crit Care Med. 2017;195(6):784-791. doi:10.1164/rccm.201604-0799OC 27635668 PMC5363967

[17] Lameire NH, Levin A, Kellum JA, ; Conference Participants. Harmonizing acute and chronic kidney disease definition and classification: report of a Kidney Disease: Improving Global Outcomes (KDIGO) consensus conference. Kidney Int. 2021;100(3):516-526. doi:10.1016/j.kint.2021.06.028 34252450

[18] Kelly YP, Waikar SS, Mendu ML. When to stop renal replacement therapy in anticipation of renal recovery in AKI: the need for consensus guidelines. Semin Dial. 2019;32(3):205-209. doi:10.1111/sdi.12773 30690779

[19] Alten JA, Cooper DS, Blinder JJ, ; Neonatal and Pediatric Heart and Renal Outcomes Network (NEPHRON) Investigators. Epidemiology of acute kidney injury after neonatal cardiac surgery: a report from the Multicenter Neonatal and Pediatric Heart and Renal Outcomes Network. Crit Care Med. 2021;49(10):e941-e951. doi:10.1097/CCM.0000000000005165 34166288

[20] Fuhrman DY, Nguyen L, Joyce EL, Priyanka P, Kellum JA. Outcomes of adults with congenital heart disease that experience acute kidney injury in the intensive care unit. Cardiol Young. 2021;31(2):274-278. doi:10.1017/S1047951120003923 33191892 PMC7897224

[21] Bagshaw SM, Wald R, Adhikari NKJ, ; STARRT-AKI Investigators; Canadian Critical Care Trials Group; Australian and New Zealand Intensive Care Society Clinical Trials Group; United Kingdom Critical Care Research Group; Canadian Nephrology Trials Network; Irish Critical Care Trials Group. Timing of initiation of renal-replacement therapy in acute kidney injury. N Engl J Med. 2020;383(3):240-251. doi:10.1056/NEJMoa2000741 32668114

[22] Li X, Liu C, Mao Z, Li Q, Zhou F. Timing of renal replacement therapy initiation for acute kidney injury in critically ill patients: a systematic review of randomized clinical trials with meta-analysis and trial sequential analysis. Crit Care. 2021;25(1):15. doi:10.1186/s13054-020-03451-y 33407756 PMC7789484

[23] Silversides JA, Pinto R, Kuint R, . Fluid balance, intradialytic hypotension, and outcomes in critically ill patients undergoing renal replacement therapy: a cohort study. Crit Care. 2014;18(6):624. doi:10.1186/s13054-014-0624-8 25407408 PMC4255668

[24] Schiffl H. Renal recovery after severe acute renal injury. Eur J Med Res. 2008;13(12):552-556. 19073394

[25] Hoste EA, Blot SI, Lameire NH, Vanholder RC, De Bacquer D, Colardyn FA. Effect of nosocomial bloodstream infection on the outcome of critically ill patients with acute renal failure treated with renal replacement therapy. J Am Soc Nephrol. 2004;15(2):454-462. doi:10.1097/01.ASN.0000110182.14608.0C 14747393

[26] Bagshaw SM, Neto AS, Smith O, ; STARRT-AKI Investigators. Impact of renal-replacement therapy strategies on outcomes for patients with chronic kidney disease: a secondary analysis of the STARRT-AKI trial. Intensive Care Med. 2022;48(12):1736-1750. doi:10.1007/s00134-022-06912-w 36331570

[27] Daverio M, Cortina G, Jones A, ; Critical Care Nephrology Section of the European Society of Paediatric and Neonatal Intensive Care. Continuous kidney replacement therapy practices in pediatric intensive care units across Europe. JAMA Netw Open. 2022;5(12):e2246901. doi:10.1001/jamanetworkopen.2022.46901 36520438 PMC9856326

[28] Gist KM, Fuhrman DY, Akcan-Arikan A. Standardizing care in pediatric continuous kidney replacement therapy-can we reach consensus without adequate evidence? JAMA Netw Open. 2022;5(12):e2246909. doi:10.1001/jamanetworkopen.2022.46909 36520442 PMC11271682

