# Supplementary material for: Major Adverse Kidney Events in Pediatric Continuous Kidney Replacement Therapy
Source: JAMA Netw Open. 2024 Feb 23;7(2):e240243. doi: 10.1001/jamanetworkopen.2024.0243 (PMC10891477; doi:10.1001/jamanetworkopen.2024.0243)
Supplement: Supplement 1. — eTable 1. Patient Outcomes Comparing 3 Liberation Patterns eTable 2. Multivariable Regression Model Estimating Persistent Kidney Dysfunction or Dialysis at 90 d eFigure 1. Kaplan-Meier Curve Summarizing Probability of Death Over 90 d for Overall Cohort eFigure 2. Estimated Probability of Meeting Primary Outcome Criteria by Liberation Pattern From Logistic Regression Model eFigure 3. Calibration Curves and Estimation Accuracy Measures for Logistic Regression Models of Primary Outcome [file jamanetwopen-e240243-s001.pdf]

Supplemental Online Content

Fuhrman DY, Stenson EK, Alhamoud I, et al; on behalf of WE-ROCK Investigators. Major adverse kidney events in pediatric continuous kidney replacement therapy. *JAMA Netw Open*. 2024;7(2):e240243. doi:10.1001/jamanetworkopen.2024.0243

eTable 1. Patient Outcomes Comparing 3 Liberation Patterns
eTable 2. Multivariable Regression Model Estimating Persistent Kidney Dysfunction or Dialysis at 90 d
eFigure 1. Kaplan-Meier Curve Summarizing Probability of Death Over 90 d for Overall Cohort
eFigure 2. Estimated Probability of Meeting Primary Outcome Criteria by Liberation Pattern From Logistic Regression Model
eFigure 3. Calibration Curves and Estimation Accuracy Measures for Logistic Regression Models of Primary Outcome

This supplemental material has been provided by the authors to give readers additional information about their work.

© 2024 Fuhrman DY et al. *JAMA Network Open.*

eTable 2: Patient Outcomes Comparing the 3 Liberation Patterns

Variable[a]	Overall N=980	Liberation Not Attempted N=357	Reinstituted N=288	Liberated N=335	*P* Value
ICU Mortality	351 (35.8)	285 (79.8)	43 (14.9)	23 (6.9)	**<.001**
Hospital Mortality	371 (37.9)	291 (81.5)	49 (17.0)	31 (9.3)	**<.001**
ICU Length of Stay (Days)[b]	22 (12-42)	44 (23-73)	24 (14-43)	18 (11-32)	**<.001**
Duration of Mechanical Ventilation (Days)[b]	3 (0-12)	6 (0-23)	3 (0-13)	0 (0-11)	**<.001**

[a]Data expressed as median (interquartile range) or N (%)
[b]Includes survivors only

© 2024 Fuhrman DY et al. *JAMA Network Open.*

eTable 3: Multivariable regression model predicting persistent kidney dysfunction or dialysis at 90 days

Variable	Reference	Contrast	aOR[a] (95%CI)
Liberation Pattern[b]	Reinstituted	Liberated	0.33 (0.22-0.51)
Liberation Pattern[b]	Not Attempted	Liberated	0.11 (0.05-0.25)
Weight (kg)	32.0	60.0	1.07 (0.91-1.27)
No Comorbidities	No	Yes	0.45 (0.27-0.74)
Primary Comorbidities: Cardiac	No	Yes	1.27 (0.82-1.97)
Primary Comorbidities: Nephrologic/Urologic	No	Yes	1.32 (0.62-2.82)
Primary Comorbidities: Immunologic	No	Yes	1.43 (0.74-2.76)
Primary Comorbidities: Oncologic	No	Yes	0.92 (0.55-1.55)
Sepsis at ICU admission	No	Yes	0.83 (0.51-1.36)
Vasopressor-Inotrope Score at CRRT Initiation	0	20	0.85 (0.7-1.03)
PELOD-2 Score at CRRT Initiation	4	9	1.03 (0.78-1.37)
Percent Cumulative Fluid Balance (ICU Admission to CRRT Initiation)	2.4	18.1	1.03 (0.91-1.17)
Time from ICU Admission to CRRT Initiation (days)	1	6.3	1.04 (0.97-1.11)
Median Calculated CRRT Dose (mL/kg/hour)	32	60.1	1.07 (0.91-1.27)
CRRT Duration (days)	3	14	1.12 (0.96-1.31)
Urine Output 24 hours prior to CRRT Initiation (ml/kg/h)	0.1	1.2	0.82 (0.67-1.01)

[a]Adjusted Odds ratio (aOR) and 95% confidence intervals (CI) obtained by logistic regression. aORs for continuous predictors scaled to reflect the interquartile range odds ratio (i.e., reference = 25th percentile, contrast = 75th percentile).
[b]The same model was rerun with each liberation pattern individually with no change in the covariate aORs.

© 2024 Fuhrman DY et al. *JAMA Network Open.*

eFigure 1: Kaplan-Meier Curve summarizing the probability of death over 90 days for the overall cohort. Shaded area denotes 95% confidence intervals.

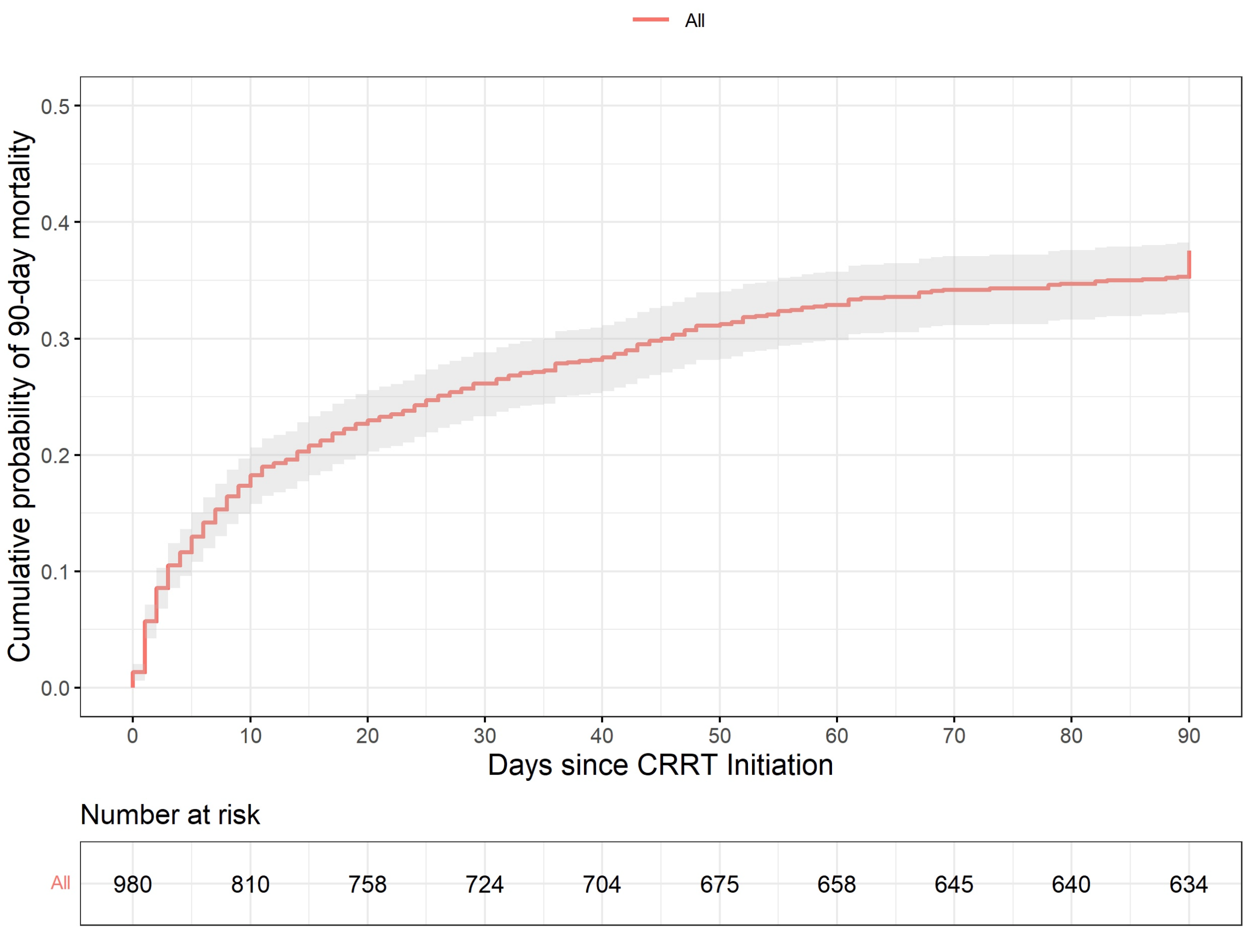

© 2024 Fuhrman DY et al. *JAMA Network Open.*

eFigure 2: **The predicted probability of meeting MAKE-90 criteria by liberation pattern from the logistic regression model**. The probability values are adjusted for weight, comorbidities, admission category, and severity of illness parameters (Vasoactive-Inotrope score and Pediatric Logistic Organ Dysfunction scores) at the time of CRRT initiation at the most frequent or median level. Error bars denote 95% confidence intervals (CI).

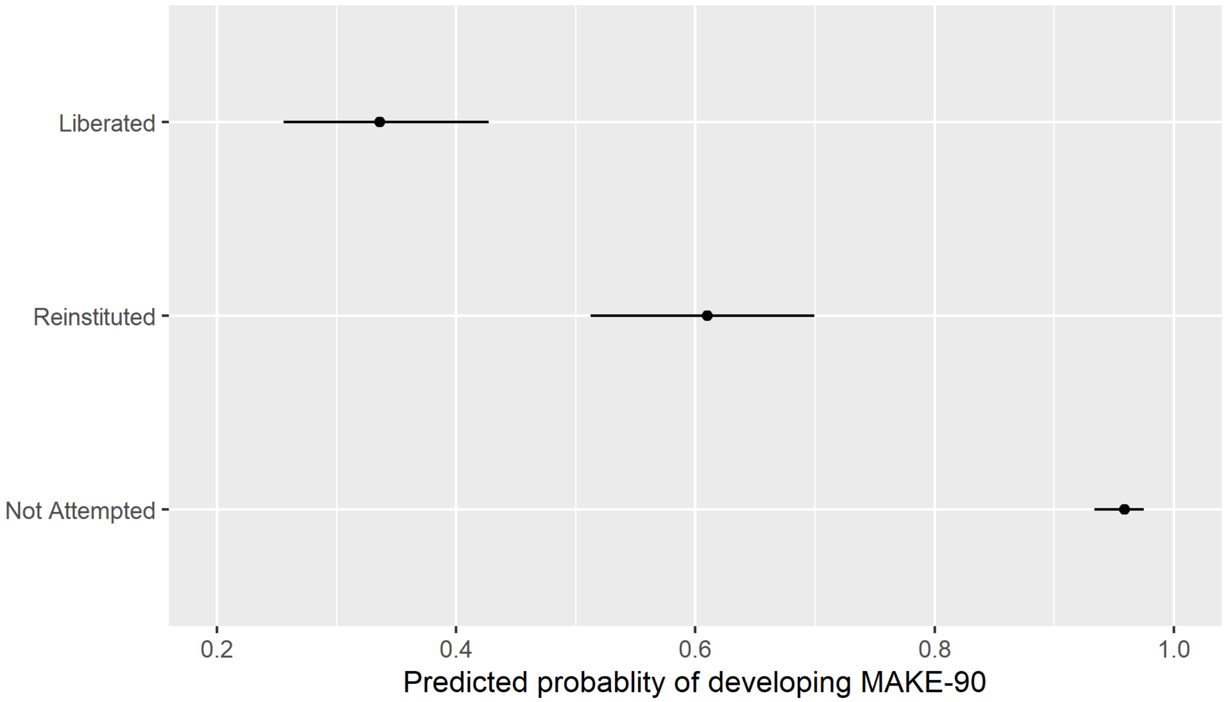

Liberation pattern	Probability of MAKE-90	95% CI
Not Attempted	95.6%	93.3%-97.5%
Reinstituted	61.0%	51.2%-69.9%
Liberated	33.6%	25.6%-42.7%

© 2024 Fuhrman DY et al. *JAMA Network Open.*

eFigure 3: **Calibration curves and predictive accuracy measures for the logistic regression models of MAKE-90.** The calibration curve is close to the ideal straight line (45 degree angle line), which indicates that the prediction results of the model are accurate and reliable.

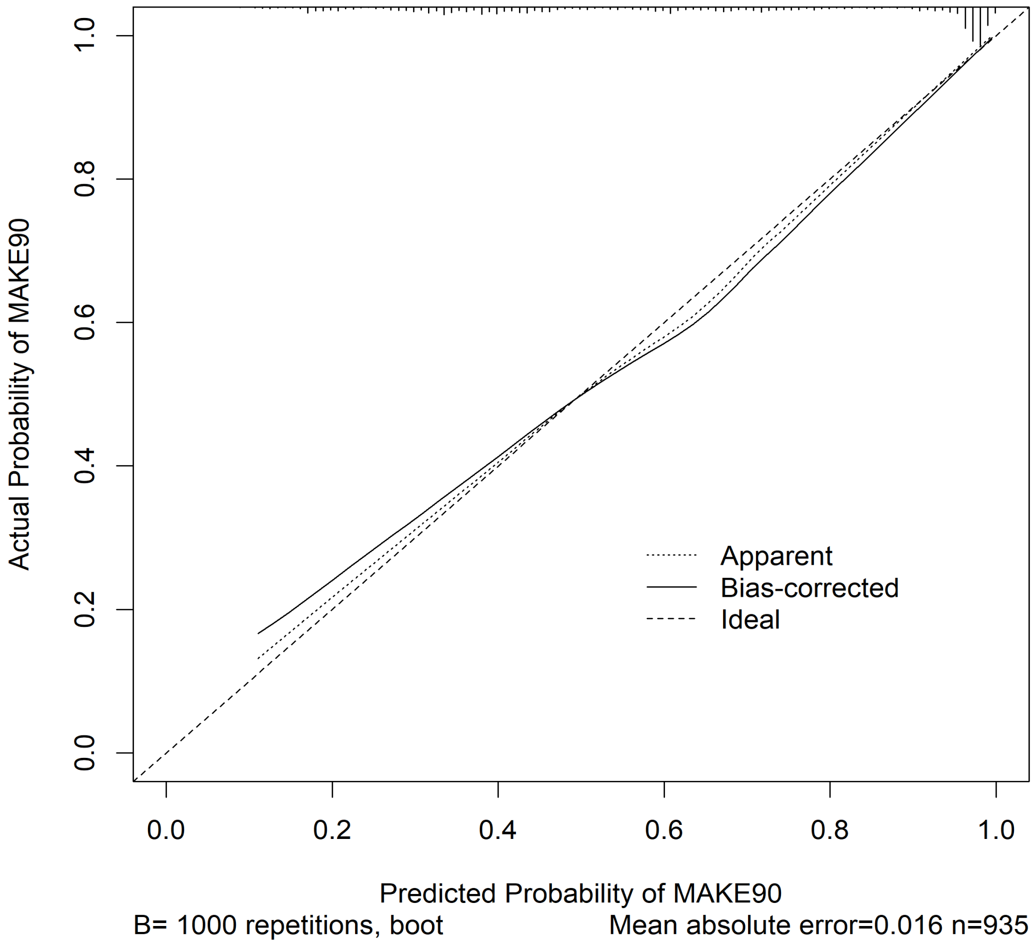

© 2024 Fuhrman DY et al. *JAMA Network Open.*
