# Supplementary material for: Major Adverse Kidney Events in Pediatric Continuous Kidney Replacement Therapy
Source: JAMA Netw Open. 2024 Feb 23;7(2):e240243. doi: 10.1001/jamanetworkopen.2024.0243 (PMC10891477; doi:10.1001/jamanetworkopen.2024.0243)
Supplement: Supplement 2. — Nonauthor Collaborators. WE-ROCK Investigators [file jamanetwopen-e240243-s002.pdf]

*Group Name: WE-ROCK Investigators							
***First Name and Middle Initial(s)**	***Last Name**	***Suffix (eg, Jr, III)**	Academic Degrees	Institution	Location (city, state/province, country)	Role or Contribution, eg, chair, principal investigator	Group (if more than 1 Group listed in the byline) and/or Subgroup (eg, Steering Committee)
Emily	Ahern		CPNP, DNP	Children's Hospital Colorado, University of Colorado School of Medicine,	Aurora, CO, USA	Data collection, manuscript review	
Ayse	Akcan Arikan		MD	Baylor College of Medicine, Texas Children's Hospital	Houston, TX, USA	Data collection, manuscript review	
Rashid	Alobaidi		MD	Univeristy of Alberta, Stollery Children's Hospital	st Louis, MO, USA	Data collection, manuscript review	
Pilar	Anton-Martin		MD, PhD	Le Bonheur Children's Hospital	Memphis, TN, USA	Dana collection, mansuript review	
Shanthi S	Balani		MD	University of Minnesota	Minneapolis, MN, USA	Data collection, manuscript review	
Matthew	Barhight		MD, MS	Ann and Robert H. Lurie Children's Hospital of Chicago	Chicago IL, USA	Data collection, manuscript review	
Abby	Basalely		MD	Cohen Children's Medical Center, Zucker School of Medicine,	Hyde Park, NY, USA	Data collection, manuscript review	
Amee	Bigelow		MD, MS	Nationwide Children's Hospital	Columbus, OH, USA	Data collection, manuscript review	
Andrea	Cappoli		MD	Bambino Gesù Children's Hospital, IRCCS	Rome, Italy	Data collection, manuscript review	
Eileen A	Ciccia		MD	Washington University School of Medicine, St. Louis Children's	St Louis, MO, USA	Data collection, manuscript review	
Michaela	Collins		BA	Cincinnati Children's Hospital Medical Center; University of Cincinnati College of Medicine	Cincinnati OH, USA	Data collection, manuscript review	
Denise	Colosimo		MD	Meyer Children's Hospital, IRCCS	Florence, Italy	Data collection, manuscript review	

***First Name and Middle Initial(s)**	***Last Name**	***Suffix (eg, Jr, III)**	Academic Degrees	Institution	Location (city, state/province, country)	Role or Contribution, eg, chair, principal investigator	Group (if more than 1 Group listed in the byline) and/or Subgroup (eg, Steering Committee)
Gerard	Cortina		MD	Medical University of Innsbruck	Innsbruck, Austria	Data collection, manuscript review	
Mihaela A	Damian		MD, MPH	Stanford University School of Medicine	Palo Alto, CA, USA	Data collection, manuscript review	
Gabrielle	DeAbreu		MD	Cohen Children's Medical Center, Zucker School of Medicine	Hyde Park, NY, USA	Data collection, manuscript review	
Akash	Deep		MD	King's College Hospital	London, England, United Kingdom	Data collection, manuscript review	
Kathy L	Ding		BS	University of Colorado, School of Medicine	Aurora, CO, USA	Data collection, manuscript review	
Kristin J	Dolan		MD	Mercy Children's Hospital and Baylor College of Medicine, Texas Children's Hospital	Kansas City, MO and Houston, TX, USA	Data collection, manuscript review	
Stephen M	Gorga		MD, MSc	University of Michigan Medical School, C.S. Mott Children's Hospital	Ann Arbor, MI, USA	Data collection, manuscript review	
Elizabeth	Harvey		MD	Hospital for Sick Children	Toronto, Ontario, Canada	Data collection, manuscript review	
Denise C	Hasson		MD	Cincinnati Children's Hospital and NYU Langone Health, Hassenfeld Children’s Hospital,	Cincinnati OH, and New York, NY, USA	Data collection, manuscript review	
Taylor	Hill-Horowitza		BS	Cohen Children's Medical Center, Zucker School of Medicine,	Hyde Park, NY, USA	Data collection, manuscript review	
Haleigh	Inthavong		BS, MS	Baylor College of Medicine, Texas Children's Hospital	Houston, TX, USA	Data collection, manuscript review	
Catherine	Joseph		MD	Baylor College of Medicine, Texas Children's Hospital	Houston, TX, USA	Data collection, manuscript review	

***First Name and Middle Initial(s)**	***Last Name**	***Suffix (eg, Jr, III)**	Academic Degrees	Institution	Location (city, state/province, country)	Role or Contribution, eg, chair, principal investigator	Group (if more than 1 Group listed in the byline) and/or Subgroup (eg, Steering Committee)
Aadil	Kakajiwala		MD	Children's National Hospital	Washington DC, USA	Data collection, manuscript review	
Aaron D	Kessel		MD, MS	Cohen Children's Medical Center, Zucker School of Medicine,	Hyde Park, NY, USA	Data collection, manuscript review	
Sarah	Korn		DO	Westchester Medical Center	Westchester, NY, USA	Data collection, manuscript review	
Kelli A	Krallman		BSN, MS	Cincinnati Children's Hospital Medical Center; University of Cincinnati College of Medicine	Cincinnati OH, USA	Data collection, manuscript review	
David M	Kwiatkowski		MD, MSc	Stanford University School of Medicine	Palo Alto, CA, USA	Data collection, manuscript review	
Jasmine	Lee		MSc	Hospital for Sick Children	Toronto, Ontario, Canada	Data collection, manuscript review	
Laurance	Lequier		MD	Univeristy of Alberta, Stollery Children's Hospital	Edmonton, Alberta, Canada	Data collection, manuscript review	
Tina	Madani Kia		BS	Univeristy of Alberta, Stollery Children's Hospital	Edmonton, Alberta, Canada	Data collection, manuscript review	
Kenneth	Mah		MD, MS	Standford School of Medicine	Palo Alto, CA, USA	Data collection, manuscript review	
Susan D	Martin		MD	Golisano Children's Hospital at University of Rochester Medical Center	Rochester, NY, USA	Data collection, manuscript review	
Shina	Menon		MD	Seattle Children's Hospital	Seattle, WA	Data collection, manuscript review	
Melissa A	Muff-Luett		MD	University of Nebraska Medical Center, Children's Hospital & Medical Center,	Omaha, NE, USA	Data collection, manuscript review	

*First Name and Middle Initial(s)	*Last Name	*Suffix (eg, Jr, III)	Academic Degrees	Institution	Location (city, state/province, country)	Role or Contribution, eg, chair, principal investigator	Group (if more than 1 Group listed in the byline) and/or Subgroup (eg, Steering Committee)
Siva	Namachivayam		MBBS	Royal Children's Hospital, University of Melbourne, Murdoch Children's Research Institute	Melbourne, Victoria, Australia	Data collection, manuscript review	
Sara	De la Mata Navazo		MD	Gregorio Marañón University Hospital School of Medicine	Madrid, Spain	Data collection, manuscript review	
Jennifer	Nhan		MD	Children's National Hospital	Washington DC, USA	Data collection, manuscript review	
Abigail	O'Rourke		MD	Cohen Children's Medical Center, Zucker School of Medicine,	Hyde Park, NY, USA	Data collection, manuscript review	
Matthew G	Pinto		MD	Maria Fareri Children's Hospital at Westchester Medical Center,	Westchester, NY, USA	Data collection, manuscript review	
Dua	Qutob		MD	Sidra Medicine and Weil Cornel Medicine	Doha, Qatar	Data collection, manuscript review	
Stephanie	Reynaud		MD	Hospital for Sick Children and Hopital Bicetre, APHP Université Paris-Saclay	Toronto, Ontario, Canada and Kremlin-Bicetre, Val de Marne, France	Data collection, manuscript review	
Zachary A	Rumlow		DO	University of Iowa Stead Family Children's Hospital, Carver College of Medicine	Iowa City, IA, USA	Data collection, manuscript review	
María J	Santiago Lozano		MD, PhD	Gregorio Marañón University Hospital; School of Medicine	Madrid, Spain	Data collection, manuscript review	
David T	Selewski		MD, MSCR	35Medical University of South Carolina	Charleston, SC, USA	Data collection, manuscript review	
Carmela	Serpe		PhD	Bambino Gesù Children's Hospital, IRCCS	Rome, Italy	Data collection, manuscript review	

*First Name and Middle Initial(s)	*Last Name	*Suffix (eg, Jr, III)	Academic Degrees	Institution	Location (city, state/province, country)	Role or Contribution, eg, chair, principal investigator	Group (if more than 1 Group listed in the byline) and/or Subgroup (eg, Steering Committee)
Alyss	Serratore		RN, MSc	Royal Children's Hospital, University of Melbourne, Murdoch Children's Research Institute	Melbourne, Victoria, Australia	Data collection, manuscript review	
Ananya	Shah		BS	University of Colorado, School of Medicine	Aurora, CO, USA	Data collection, manuscript review	
Weiwen V	Shih		MD	University of Colorado, School of Medicine	Aurora, CO, USA	Data collection, manuscript review	
H Stella	Shin		MD	Children's Healthcare of Atlanta	Atlanta, GA, USA	Data collection, manuscript review	
Cara L	Slagle		MD	Cincinnati Children's Hospital Medical Center; University of Cincinnati College of Medicine	Cincinnati OH, USA	Data collection, manuscript review	
Sonia	Solomon		DO	Westchester Medical Center, Westchester	Westchester, NY, USA	Data collection, manuscript review	
Danielle E	Soranno		MD	Indiana University School of Medicine, Riley Hospital for Children	Indianapolis, IA, USA	Data collection, manuscript review	
Rachana	Srivastava		MD	Mattel Children's Hospital at UCL	Los Angeles, CA, USA	Data collection, manuscript review	
Michelle C	Starr		MD, MPH	Indiana University School of Medicine, Riley Hospital for Children	Indianapolis, IA, USA	Data collection, manuscript review	
Amy E	Strong		MD, MSCE	University of Iowa Stead Family Children's Hospital, Carver College of Medicine	Iowa City, IA, USA	Data collection, manuscript review	
Susan A	Taylor		MSc	King's College Hospital	London, England, United Kingdom	Data collection, manuscript review	

***First Name and Middle Initial(s)**	***Last Name**	***Suffix (eg, Jr, III)**	Academic Degrees	Institution	Location (city, state/province, country)	Role or Contribution, eg, chair, principal investigator	Group (if more than 1 Group listed in the byline) and/or Subgroup (eg, Steering Committee)
Sameer V	Thadani		MD	Baylor College of Medicine, Texas Children's Hospital	Houston, TX, USA	Data collection, manuscript review	
Amanda M	Uber		DO	University of Nebraska Medical Center	Omaha, NE, USA	Data collection, manuscript review	
Brynna	Van Wyk		ARNP, MSN	University of Iowa Stead Family Children's Hospital, Carver College of Medicine	Iowa City, IA, USA	Data collection, manuscript review	
Tennille N	Webb		MD, MSPH	Children's of Alabama/University of Alabama at Birmingham	Birmingham, AL, USA	Data collection, manuscript review	
Michael	Zappitelli		MD, MSc	Hospital for Sick Children	Toronto, Ontario, Canada	Data collection, manuscript review	
Michelle C	Starr		MD, MPH	Indiana University School of Medicine, Riley Hospital for Children	Indianapolis, IA, USA	Data collection, manuscript review	
Amy E	Strong		MD, MSCE	University of Iowa Stead Family Children's Hospital, Carver College of Medicine	Iowa City, IA, USA	Data collection, manuscript review	
Susan A	Taylor		MSc	King's College Hospital	London, England, United Kingdom	Data collection, manuscript review	
Sameer V	Thadani		MD	Baylor College of Medicine, Texas Children's Hospital	Houston, TX, USA	Data collection, manuscript review	
Amanda M	Uber		DO	University of Nebraska Medical Center	Omaha, NE, USA	Data collection, manuscript review	
Brynna	Van Wyk		ARNP, MSN	University of Iowa Stead Family Children's Hospital, Carver College of Medicine	Iowa City, IA, USA	Data collection, manuscript review	

***First Name and Middle Initial(s)**	***Last Name**	***Suffix (eg, Jr, III)**	Academic Degrees	Institution	Location (city, state/province, country)	Role or Contribution, eg, chair, principal investigator	Group (if more than 1 Group listed in the byline) and/or Subgroup (eg, Steering Committee)
Tennille N	Webb		MD, MSPH	Children's of Alabama/University of Alabama at Birmingham	Birmingham, AL, USA	Data collection, manuscript review	
Emily E	Zangla		DO	University of Minnesota	Minneapolis, MN, USA	Data collection, manuscript review	
Michael	Zappitelli		MD, MSc	Hospital for Sick Children	Toronto, Ontario, Canada	Data collection, manuscript review	
