# Supplementary material for: Major Adverse Kidney Events in Pediatric Continuous Kidney Replacement Therapy
Source: JAMA Netw Open. 2024 Feb 23;7(2):e240243. doi: 10.1001/jamanetworkopen.2024.0243 (PMC10891477; doi:10.1001/jamanetworkopen.2024.0243)
Supplement: Supplement 3. — Data Sharing Statement [file jamanetwopen-e240243-s003.pdf]

Data Sharing Statement

Fuhrman. Major Adverse Kidney Events in Pediatric Continuous Kidney Replacement Therapy. *JAMA Netw Open*. Published online February 23, 2024. doi:10.1001/jamanetworkopen.2024.0243

Data
Data available: No

Additional Information
Explanation for why data not available: Individuals interested in acquiring data can apply through werockstudy.org.
